# High Hydrostatic Pressure (HHP)-Induced Structural Modification of Patatin and Its Antioxidant Activities

**DOI:** 10.3390/molecules22030438

**Published:** 2017-03-10

**Authors:** Rizwan Elahi, Tai-Hua Mu

**Affiliations:** Laboratory of Food Chemistry and Nutrition Science, Institute of Food Science and Technology, Chinese Academy of Agricultural Sciences, Ministry of Agriculture, No. 2 Yuan Ming Yuan West Road, Haidian District, Beijing 100193, China; malikbiochemist@live.com

**Keywords:** potato patatin, high hydrostatic pressure, antioxidant activities, iron chelation potential, thermal properties, surface hydrophobicity

## Abstract

Patatin represents a group of homologous primary storage proteins (with molecular weights ranging from 40 kDa to 45 kDa) found in *Solanum tuberosum* L. This group comprises 40% of the total soluble proteins in potato tubers. Here, patatin (40 kDa) was extracted from potato fruit juice using ammonium sulfate precipitation (ASP) and exposed to high hydrostatic pressure (HHP) treatment (250, 350, 450, and 550 MPa). We investigated the effect of HHP treatment on the structure, composition, heat profile, and antioxidant potential, observing prominent changes in HHP-induced patatin secondary structure as compared with native patatin (NP). Additionally, significant (*p* < 0.05) increases in β-sheet content along with decreases in α-helix content were observed following HHP treatment. Thermal changes observed by differential scanning calorimetry (DSC) also showed a similar trend following HHP treatment; however, the enthalpy of patatin was also negatively affected by pressurization, and free sulfhydryl content and surface hydrophobicity significantly increased with pressurization up to 450 MPa, although both interactions progressively decreased at 550 MPa. The observed physicochemical changes suggested conformational modifications in patatin induced by HHP treatment. Moreover, our results indicated marked enhancement of antioxidant potential, as well as iron chelation activities, in HHP-treated patatin as compared with NP. These results suggested that HHP treatment offers an effective and green process for inducing structural modifications and improving patatin functionality.

## 1. Introduction

Potato (*Solanum tuberosum* L.) is the most important non-grain food crop in the world and is central to global food security. The potato is a member of the Solanaceae family, which includes tomatoes, peppers, aubergines (eggplants), petunias, and tobacco. According to recent Food and Agriculture Organization estimates, China is the biggest global potato producer, with 87.26 million metric tons which constitutes almost one-third of the total worldwide production [1]. The rapidly growing demand for plant-based proteins due to their economic affordability and relatively higher nutritional values, including attractive amino acid profile, low caloric content, and ease of digestibility, has replaced that for expensive animal proteins [2].

Soluble proteins present in potato fruit juice (PFJ) have been divided into three groups: patatins (30%–40%), protease inhibitors (40%–50%), and other proteins (10%–15%) (mainly enzymes, such as kinases and enzymes involved in starch synthesis) [3,4,5,6,7,8]. Patatin represents a group of immunologically identical glycoprotein isoforms with a monomeric molecular weight (MW) from ~40 kDa to 45 kDa (the native conformation is a dimer) [9] and isoelectric point value between 4.5 and 5.2 [10]. It was first purified by Racusen and Foote [9] using diethylaminoethyl (DEAE)-cellulose and concanavalin A-sepharose chromatography and comprises ~40% of the total soluble protein of a mature potato tuber. As a glycoprotein, it contains monosaccharide residues, including mannose, galactose, glucose, and rhamnose, connected as α-mannose (1→3), α-galactose (1→4), β-glucose (1→4), and α-rhamnose (1→2) linkages, respectively [11].

Existing methods for extracting potato-protein isolates include ultrafiltration, ion exchange, gel permeation, affinity and mixed-mode chromatography, carboxymethyl cellulose complexation, acid and heat coagulation, and ethanol and various salt-precipitation methods [12,13]. (NH_4_)_2_SO_4_ precipitation is one of the most stable methods used to isolate high-yield patatin exhibiting better physicochemical and structural characteristics [13].

The globular structure of patatin offers versatile bioactive sites for numerous biofunctionalities, such as antioxidant, enzymatic, and anticancer activities [11,14,15,16,17]. Patatin isoforms at 45 kDa exhibit antioxidant activity, such as 1,1-diphenyl-2-picrylhydrazyl (DPPH)-radical-scavenging activity, anti-human low-density lipoprotein peroxidation, protection against hydroxyl radical-mediated DNA damage, and peroxynitrite-mediated dihydrorhodamine 123 oxidation, based on a series of in vitro assays. Moreover, recent studies by Sun et al. [11] reported an antioxidant role for patatin according to its various antioxidant activities. Unlike classical lipases, the unique structural topology of patatin enables it to act as a lipid hydrolase [18].

Various biologically active peptides are usually inactive within the sequence of native proteins; therefore, modification via several means (physical, chemical, or enzymatic,) is required to improve their biological activities. Recently, several studies focused on eco-friendly techniques, such as the use of high hydrostatic pressure (HHP), which is relatively more economical and effective [19]. HHP is an alternative, non-thermal, food-processing method showing promise for the development of new food products exhibiting additional functional and health benefits [20]. HPP is an applied technique already successfully utilized in mostly protein-product industries [21]. Pressure-induced changes at less than between 100 MPa and 250 MPa are sometimes reversible and temporary, whereas those >300 MPa cause irreversible conformation changes [22]. HHP affects only non-covalent bonds (hydrogen, ionic, and hydrophobic bonds) and alters the protein structure by unfolding protein chains [23]. According to the United States Food and Drug Administration, potato proteins are intended for use in a variety of protein-related functions, including as water binders in meat and sausage, foaming aids in confectionary, bakery, and dairy products, and as emulsifiers in spreads, sauces, desserts, and dressings. The described uses of coagulated potato protein and hydrolyzed coagulated potato protein mark them as generally recognized as safe.

Here, we isolated and purified patatin to a high purity from *S. tuberosum* L. and evaluated the effect of HHP on its secondary structure and thermal stability in order to compare the functional properties of NP and HHP-treated patatin regarding their antioxidant potential and iron-chelating activities.

## 2. Results and Discussion

### 2.1. Patatin Purification

**Figure 1:**
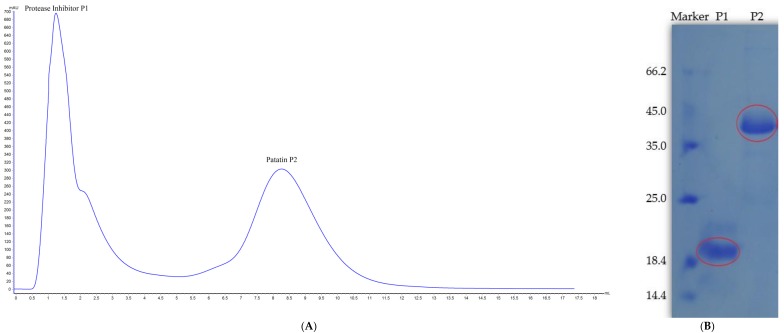
(**A**) Patatin purification using AKTA Protein Pure M1 and a HiTrap DEAE-sepharose FF (1 mL); (**B**) SDS-PAGE results showing peak 1 at MW ~20 kDa representing the protease inhibitor and peak 2 at ~40 kDa representing patatin.

**Figure 2:**
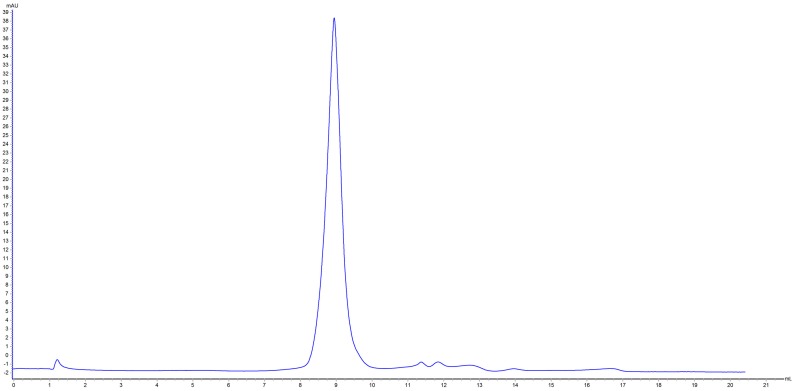
Superdex 200 10/300 gel filtration chromatograph of peak 2 results from ion-exchange chromatography.

Patatin is slightly acidic, although at neutral pH and ambient temperature it exists as a dimer held together by non-covalent hydrophobic forces [24]. Two peaks were obtained following potato-protein elution and confirmed by SDS-PAGE analysis (Figure 1). Peak 1 showed a MW of ~20 kDa representing a protease inhibitor, with peak 2 at 40 kDa representing patatin. Based on the MW difference, the 40-kDa peak was further purified by gel filtration chromatography to obtain high-purity patatin (Figure 2). Subsequently, purified patatin was dialyzed (MWCO 12–14 kDa; P. Inter-trade Equipment, Seguin, TX, USA) against distilled water at 4 °C until the conductivity of the retentate reached ≤2 μs·cm^−1^ and remained constant. Dialysis through a semipermeable membrane is among the most convenient methods for removing salts or other small molecules following purification. Dialyzed samples were removed from the membrane and stored at −20 °C following lyophilization.

### 2.2. Patatin Yield and Purity

The protein content of lyophilized patatin as determined using the Kjeldahl method [25] was 89.21%, with a protein-nitrogen coefficient of 6.25. Total soluble protein was previously determined by Lowry et al. [26] using bovine serum albumin as the standard calibration curve with a linearity range R^2^ = 0.998. Results indicated total protein content 96.218 µg·mL^−1^.

### 2.3. Effects of HHP on MW Distribution of Patatin

**Figure 3:**
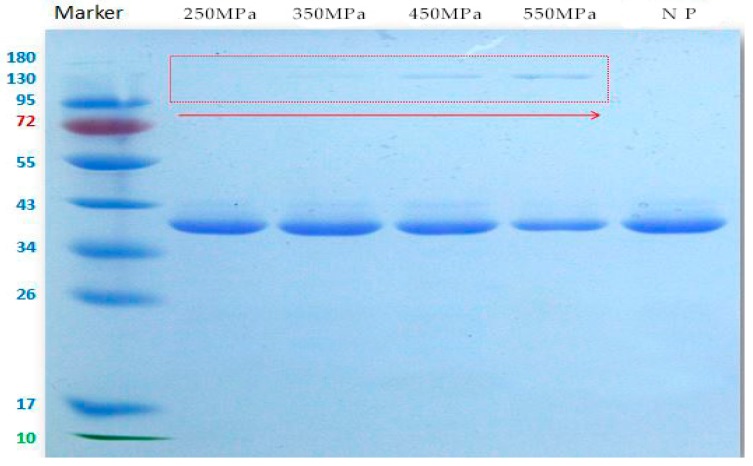
SDS-PAGE results showing NP and HHP-modified patatin under reducing conditions.

As shown in Figure 3, a visible band appeared at MW > 40 kDa in each NP and HHP-treated sample, agreeing with previous results describing purified patatin [9,10,27,28]. HHP-treated patatin (250–550 MPa) showed a clearly increasing pattern of new protein bands at MW > 130 kDa, which could be due to HPP-induced aggregation of patatin. Intensity bands were directly proportional to the increase in pressure treatment as depicted in SDS-PAGE gel. Similar findings were previously reported from rapeseed, soy, and amaranth proteins [29,30,31], and one report related this aggregation to the intermolecular disulfide bond formation as a result of pressurization [32]. The absence of a band at ~130 kDa for the NP protein clearly indicated that aggregation occurred due to HHP treatment.

### 2.4. Neutral Sugar Composition

Due to the glycoprotein composition of patatin, studies revealed that ~6% of patatin consists of neutral sugar and hexosamine [6,33]. Usually, patatin consists of arabinose, rhamnose, galactose, glucose, and xylose at different ratios dependent upon the variety and extraction method used [3,34]. Results showed a significant increase in galactose content (18.04% ± 0.57% and 17.03% ± 1.13% at 250 MPa and 550 MPa, respectively, Table 1), whereas a subsequent decrease in glucose content was also observed following HHP treatment (10.93% ± 0.24%, 10.70% ± 0.08%, 12.30% ± 0.25%, and 11.24% ± 0.37% at 250 to 550 MPa, respectively).

This might be explained by the interconversion of glucose into galactose, whereas a lower effect of HHP treatment was observed on xylose and mannose content. This was likely due to application of high pressure unfolding the protein secondary structure, thereby detaching some of the bound neutral sugar groups from the protein.

### 2.5. FTIR Analysis

**Figure 4:**
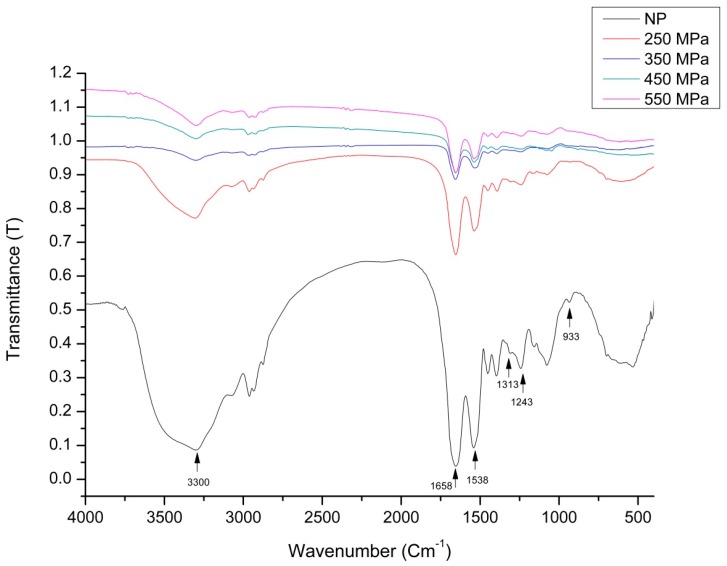
FTIR spectra of NP and HHP-treated patatin.

FTIR spectra depicted pressure-induced changes in the patatin secondary structure according to characteristic shifts in several band frequencies (Figure 4). The results also showed significant increases in transmittance intensity along with increases in pressure from 250 MPa to 550 MPa, with NP showing the least intensity at 0.52 for 250 MPa and maximum intensity at 1.15 for 550. Based on the peak intensities, these findings suggested that interchain interactions increased along with increasing pressure [35].

Both the NP and HHP-induced patatin samples returned a single major band at 1658 cm^−1^ and 1538 cm^−1^ associated with amide I and amide II bands, respectively. The spectral shapes of amide I (1600–1700 cm^−1^) and amide II (1500–1550 cm^−1^) did not change considerably when treated at a pressure level of 250 MPa. The absorption associated with the amide I band was due to stretching vibrations of the C=O amide bond and reflects the secondary structure of the protein. However, HHP-induced modifications led to a pronounced decrease in intensity of this characteristic peak. Based on the pressure effects on the widths of the depolarized and weakly polarized bands, the reorientation rate of the chains appeared dampened along with increasing pressure. The bandwidth of the strongly polarized bands (symmetric CH_2_-stretching) was an indication of interchain interactions [35]. Changes in the secondary structures (loss of intensity) of highly pressurized proteins (>350 MPa) were observed at ~1658 cm^−1^ and 1538 cm^−1^ (Figure 4). These frequencies correspond to β-structures, which were significantly affected by HHP treatment. Additionally, obvious decreases in the intensity of bands at 1540 cm^−1^ in modified proteins suggested denaturation of the α-helix, which were similar to patterns of change reported by Li et al. [36]. For the untreated sample, a strong band was noted at 1658 cm^−1^, which likely corresponded to an α-helix. This band in the HHP-treated samples was diminished, indicating that the α-helix was altered. A strong band was observed at 3300 cm^−1^ due to amide stretching in proteins, which was significantly reduced following HHP treatment. HHP-induced patatin also showed similar spectra exhibiting decreased intensity as compared to those for NP patatin. NP presented a peak at 1389 cm^−1^, indicating an absorbance of C–O stretching typically at ~1400 cm^−1^ to 1330 cm^−1^ [37]. An important band associated with secondary structure represents C–C stretching regions (890–1060 cm^−1^). We observed a band in NP at 933 cm^−1^, which reduced abruptly in all HHP-treated samples. Our findings agreed with those of Hoppe [20], who reported marked reductions in α-helices and prominent increases in β-sheets following increased HHP treatment. There results indicated that application of HPP up to 550 MPa induced changes in patatin secondary structure.

### 2.6. DSC

Patatin thermal stability as measured by DSC and influenced by HHP is shown in Table 2. Our results shows that NP exhibited a Td_2_ of 66.62 °C and a ΔH of 24.03 J·g^−1^. As shown in Table 2, a slight decrease in T_d2_ from 65.88 °C to 65.64 °C was observed in patatin samples treated with 250 MPa to 550 MPa, respectively, indicating low-affinity protein-protein interactions as a result of aggregation [29]. The ΔH of the HHP-treated patatin showed a significant (*p* < 0.05) reduction from 24.03 J·g^−1^ to 3.056 J·g^−1^ with increased pressurization from 250 MPa to 550 MPa. These findings agreed with those previously reported for rapeseed protein and soy protein, with decreases in ΔH from 10.25 J·g^−1^ to 3.72 J·g^−1^ and 7.8 J·g^−1^ to 0.6 J·g^−1^, respectively [29,38]. These thermal parameters may be a useful guide in the design of appropriate HHP schemes for application and incorporation of patatin in food and nutraceutical products.

### 2.7. CD

Results presented in Table 3 show significant increases in β-sheet conformations along with elevations in HHP (NP (24.2%), 250 MPa (26.5%), 350 MPa (31.6%), 450 MPa (36.4%), and 550 MPa (39.5%)), whereas decreased α-helix conformations was observed at the same pressures (24.2%, 21.7%, 16.3%, 7.3%, and 4.1%, respectively). High-pressure treatment influenced the intermolecular and intramolecular attractions in unfolded protein structures by increasing β-sheet conformations and reductions in α-helices, which agreed with previously reported literature [39,40,41,42].

Decreases in α-helix conformation and random-coil structures along with increased β-sheet conformations induced by HHP treatment from 250 MPa to 550 MPa might result from the formation of intermolecular hydrogen bonding, which ultimately results in insoluble protein aggregates that modify secondary structure [43].

### 2.8. H_o_

**Figure 5:**
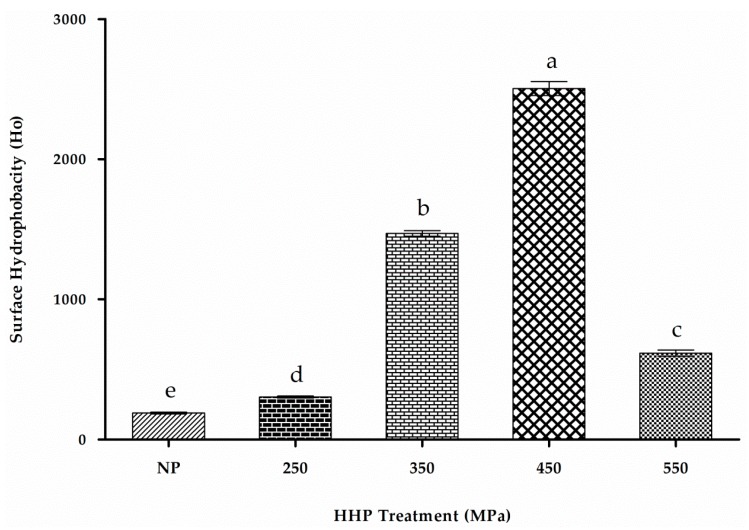
Flourometric results of NP and HHP-modified patatin (pH 7.0) measured at 390 nm (excitation), with emission measured between 300 nm and 800 nm. Characters (a–e) on the top of each column indicate significant differences (*p* < 0.05). Each data point represents the mean ± SD of triplicate treatments.

H_o_ has greater significance in elucidating protein function associated with biological phenomena [44]. ANS was used as a hydrophobic fluorescent probe due to its increased specificity toward hydrophobic sites of protein molecules. Changes in hydrophobicity promoted by structural and conformational changes in proteins allow different molecules to interact, ultimately resulting in potentially improved activity. Changes in patatin H_o_ were induced by HHP probed by ANS as indicated following ANS binding to proteins via electrostatic interactions, resulting in fluorescence signals upon binding to hydrophobic domains. Fluorescence spectra are mainly attributed to tryptophan, tyrosine, and phenylalanine residues, with tryptophan specifically contributing to the fluorescence quantum yield, which decreases as their exposure to solvent increases [45]. As shown in Figure 5, HHP treatment resulted in significant effects on the molecular properties of patatin (*p* < 0.05), with increases in H_o_ (289.8 ± 1.2, 1438 ± 1.6, and 2434.7 ± 2.4) at 250, 350 and 450 MPa respectively, as compared with those observed in NP (H_o_ = 180.4 ± 1.5).

While a dramatic drop (H_o_ = 587.5 ± 1.8) was observed when treated at 550 MPa, the gradual increase in H_o_ in HHP-treated samples might be attributed to the increased exposure of buried nonpolar (hydrophobic sites), which were ultimately bound to the ANS probe and resulted in increased fluorescence. The lower S_0_ values in NP might be attributed to the unexposed hydrophobic groups becoming buried and less accessible to ANS. The highest H_o_ value occurred at 450 MPa (H_o_ = 2434.7 ± 2.4), which might be explained by protein dissociation, leading to the exposure of additional hydrophobic regions. These results are in agreement with previous findings reporting HHP-induced hydrophobicity in different proteins [38,46]. Furthermore, a previous report showed that a gradual increase to 400 MPa and ultimately reaching 600 MPa significantly reduced the hydrophobicity of soy protein [47]. Additionally, ANS as an anionic probe could easily interact with positively charged sites on HHP-modified patatin, thereby resulting in potential overestimation of hydrophobicity [48], although the H_o_ values determined by the same ANS probe were slightly higher than those derived from lentil legumin-like protein [49] and other proteins from peas and soybeans [47,49]. The FTIR spectra also confirmed the findings of elevated hydrophobicity due to changes in CH-stretching and the amide III band [20]. These results indicated that the globular confirmation of NP consists of a compact folded structure, and that pressurization unfolds it to allow a more open and flexible conformation, ultimately exposing additional hydrophobic regions and increasing overall hydrophobicity [50]. The NP is usually folded sufficiently to bury most of the hydrophobic amino acids, which may hinder the exposure of hydrophobic regions. HHP treatment might have exposed the buried hydrophobic residues to enhance surface hydrophobicity. Our findings suggested that patatin is more sensitive to HHP treatment than other storage proteins.

### 2.9. Free-SH

**Figure 6:**
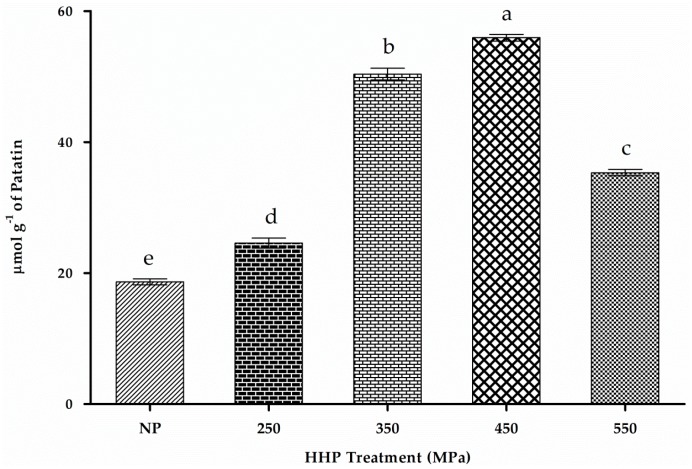
Free-SH content of NP and HHP-treated patatin (250–550 MPa). Characters (a–e) on the top of each column indicate significant differences (*p* < 0.05) difference among the groups. Results represent the mean ± SD of triplicate treatments.

Positive and negative charges of side chains are randomly distributed over the patatin sequence, and it contains one cysteine residue [51]. Figure 6 shows a significant increase (*p* < 0.05) in free-SH content (23.2 ± 0.34, 52.0 ± 0.8 and 55.6 ± 0.2 μmol·g^−1^) along with increased HHP treatment at 250, 350 and 450 MPa respectively.

Increases in free-SH content in the presence of HHP at up to 450 MPa might be attributed to the pressure-induced exposure of embedded SH groups inside the structure or cleavage of S-S linkage. Usually, 213.1 kJ·mol^−1^ of energy is required to disrupt disulfide bonds above the normal HHP-treatment ranges (~600 MPa). Therefore, covalent peptide linkages usually remain unaffected by HHP treatment [52]. The marked increase in free-SH groups might be related to the unfolding of the protein following exposure of embedded SH groups [53]. Previous findings on amaranth and rapeseed protein isolates showed similar trends [29,31]. We observed abrupt decreases in free-SH content upon increasing the pressure to 550 MPa (34.4 ± 0.8 μmol·g^−1^), which might be due to re-arrangement of free-SH in other peptide regions to form S-S linkages leading to protein aggregation [29,38,54]. Previous studies also reported that this trend might be due to protein aggregation and cross-linking through intermolecular interaction [55]. SDS-PAGE results (Figure 3) confirmed this phenomenon of newly formed S-S bonds, which appeared to contribute to high-MW fractions in HHP-treated samples due to aggregation [38]. Formation of high-MW aggregates through the formation of disulfide bonds in α- and β-lactoglobulin following HHP treatment at >200 MPa was previously reported [38,54]. Other reports concerning whey protein indicated that free-SH groups might contribute to antioxidative activities [56]. Decreases in free-SH groups at 550 MPa might also be due to increased pressurization forcing reformation of disulfide bonds between free-SH groups [53]. However, amaranth protein showed a continuous increase in free-SH content in the presence of increasing pressure up to 600 MPa [31]. It is assumed that the differences in structural and conformational properties of proteins are reflected in their behaviors to HPP treatment. These results confirmed the occurrence of HPP-induced protein unfolding and subsequent aggregation/re-association of the unfolded proteins.

### 2.10. DPPH-Radical-Scavenging Activity

**Figure 7:**
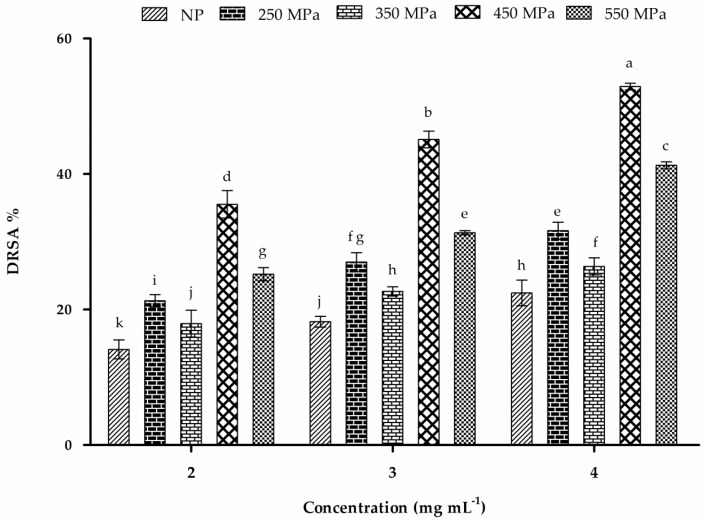
DPPH-radical-scavenging activity of NP and HHP-treated patatin at different concentrations. Characters (a–k) on the top of each column indicate significant differences (*p* < 0.05). Each data point represents the mean ± SD of triplicate treatments.

The results of DPPH-radical-scavenging assays are shown in Figure 7. Our findings revealed that antioxidant activity was markedly enhanced following HHP treatment. Maximum activity (52.8% ± 0.5%) was observed with patatin treated with 450 MPa at a concentration of 4 mg·mL^−1^. Liu et al. [17] reported that thiol groups of cysteine and tryptophan residues in patatin might have major contribution in radical scavenging activity. The IC_50_ value for patatin (45 kDa) associated with DPPH-radical-scavenging activity at 0.582 mg·mL^−1^ [17], was significantly higher than that of patatin (40.6 kDa) reported by Sun et al. [11]. This difference in antioxidant potential might be due to different patatin isoforms, as well as differences in extraction method, which would ultimately result in variable bioactivities. Trypsin inhibitor from sweet potato (33 kDa) exhibited profound scavenging activity (22% at 46.8 pmol) against DPPH radical, although it was suggested that free-SH groups in the trypsin inhibitor might be responsible for these antioxidant activities [57].

HHP treatment at 250 MPa resulted in relatively fewer conformational changes and less functional activity as compared with those observed at higher pressure ranges. This could be attributed to the reversible behavior of the protein due to its elasticity and weak interactions between intermolecular and intramolecular forces [58]. These results indicated that HHP treatment might be an effective method to enhance the antioxidant activity of patatin.

### 2.11. ORAC

**Figure 8:**
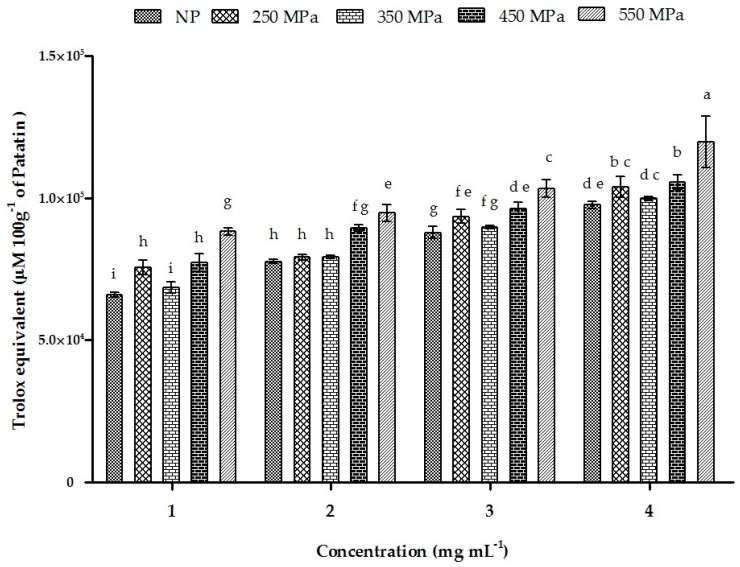
The ORAC activity of NP and HHP-treated patatin. Characters (a–i) on the top of each column indicate significant differences (*p* < 0.001) among the groups. Each data point represents the mean ± SD of triplicate treatments.

The ORACs of NP and HHP-treated patatin are presented in Figure 8. The Trolox-standard curve was plotted between 0 µg·mL^−1^ and 60 µg·mL^−1^, with a Net_AUC_ standard equation of *Y* = 0.899*x* + 2.581 (R^2^ = 0.993). We observed a strong correlation between patatin concentration and antioxidant potential for the NP protein and patatin treated with 250 MPa, 350 MPa, 450 MPa, and 550 MPa. Patatin pressurized at 550 MPa showed maximum activity at 4 mg·mL^−1^ and yielded 110,700 ± 106 µM TE 100 g^−1^, followed by results from pressurization at 450 MPa (105,688 ± 208 µM TE 100 g^−1^), which was relatively higher than results for NP patatin (99,860 ± 56.1 µM TE 100 g^−1^). Treatment at 250 MPa resulted in higher TE values in a dose-dependent manner, which might be attributed to the increased availability of the patatin active site following pressurization. Comparison of patatin results with those of other Solanaceae family members revealed that NP exhibited a higher ORAC value as compared with tomato and eggplant, but lower than that of pepper, thereby indicating that it might be a more effective antioxidant relative to some family members [59].

### 2.12. Iron-Chelating Activity

**Figure 9:**
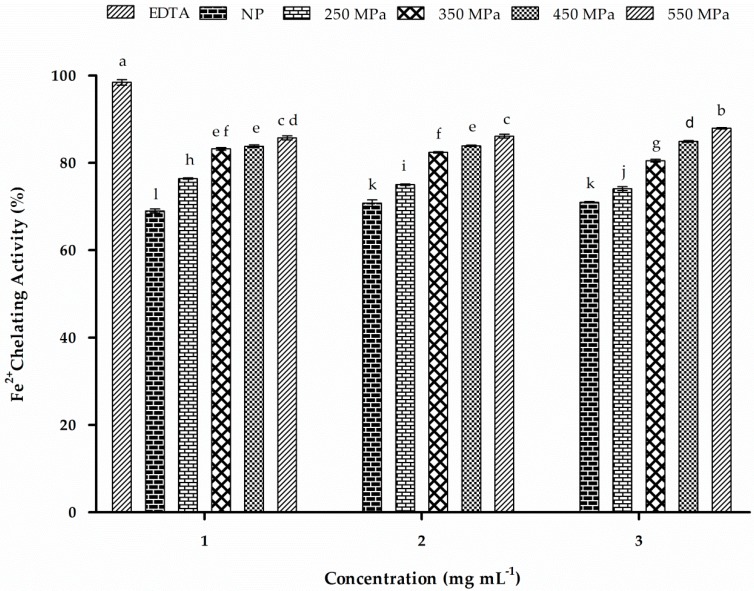
Fe^2+^-chelating activity at different concentrations (1, 2, and 3 mg·mL^−1^). EDTA was used as the positive control. Characters (a–l) on the top of each column indicate significant differences (*p* < 0.05) among the groups. All results represent the mean ± SD of each value.

Inhibition of ferrozine-Fe^2+^ complex formation in the presence of chelating agents is indicated by reduced coloration of the reaction mixture. Several proteins from plants, as well as their hydrolysates, were studied to explore their metal chelating abilities [60]. Both NP and HHP-treated patatin showed significant (*p* < 0.05) iron-chelating activity (Figure 9). All HHP-treated patatin samples showed improved Fe^2+^-chelation ability as compared with that observed by NP patatin. HHP treatment at 550 MPa resulted in the highest activity (87.7% at 3 mg·mL^−1^), followed by 450 MPa (84.8%), 350 MPa (82.4%), and 250 MPa (76.2%). However, at the same concentration, the NP showed relatively lower chelation ability (71.8%). Although both NP and HHP-treated patatin showed chelation activity >50% at all concentrations, dose-dependent effects were not significant from 1 mg·mL^−1^ to 3 mg·mL^−1^. Our findings indicated improved Fe^2+^-chelating activity in patatin as compared with that observed in other plant proteins, such as cannabis protein hydrolysates (50% at 2.2 mg·mL^−1^), sweet potato protein hydrolysate (34.74% at 2 mg·mL^−1^), and whey protein hydrolysate (<60% at 8% protein) [61,62]. However, gram wheat protein isolate showed better Fe^2+^-chelating activity at ~89% at 1 mg·mL^−1^ [60]. These results indicated that patatin exhibited improved iron-binding ability following HHP treatment, suggesting that the iron-chelating ability of patatin might be important in its role as an antioxidant.

## 3. Materials and Methods

### 3.1. Chemicals and Reagents

1,1-Diphenyl-2-picrylhydrazyl free radical (DPPH) free radical (DPPH), trolox (6-hydroxy-2,5,7,8-tetramethethylchroman-2-carboxylic acid), 2,4,6-tris(2-pyridyl)-s-triazine) (TPTZ) and 2,2′-azobis (2-aminopropane) dihydrochloride (AAPH) were obtained from Sigma-Aldrich (St. Louis, MO, USA). DEAE-sepharose fast flow and superdex 200 10/300 were obtained from (GE Healthcare BioScience, Stockholm, Sweden). All other chemicals used in this study were of analytical grade unless otherwise stated.

### 3.2. Patatin Isolation and Purification

Patatin was isolated from potatoes according to the procedure of Racusen and Foote [6] with slight modifications. Initially, fresh potatoes (*S. tuberosum* L.) were purchased from the local market and stored at 4 °C. Potatoes were carefully sorted, washed, and peeled, followed by chopping (1 × 1 × 5 cm). Chopped potatoes were kept in 50 mM NaHSO_3_ to prevent enzymatic browning. PFJ was extracted using a domestic juice extractor (HR1866/30; Philips, Eindhoven, The Netherlands), filtered through a 120-mm mesh sieve, and the starch slurry was allowed to sediment. The PFJ was centrifuged twice at 3000× *g* for 10 min (LXJ-IIC; Shanghai Anting Scientific Instruments, Shanghai, China) to remove all traces of starch from the juice.

Ammonium sulfate precipitate (ASP) was prepared from PFJ by adding (NH_4_)_2_SO_4_ to 60% saturation as described by Seppala et al. [63] and maintaining the pH at 5.7 by addition of small volumes of 0.5 M H_2_SO_4_. After 1 h at 4 °C, the suspension was centrifuged (30 min at 19,000× *g* at 4 °C), and the resulting precipitate was washed twice with half of the starting volume of 50 mM sodium phosphate buffer (pH 7.0) containing (NH_4_)_2_SO_4_ up to 60% saturation. Subsequently, the protein isolate was filtered using an ultrafiltration (UF) column with a MW cut-off of 20 kDa and a reverse osmosis cellulose acetate membrane (Shenzhen Feyian Water Treatment Technology Co., Ltd., Shenzhen, China) at 4 °C to remove low MW compounds and salts. The retentate was subsequently freeze-dried and stored at −20 °C. Additional patatin purification was performed using an ÄKTA Protein Pure M1 system with unicorn software and HiTrap DEAE-sepharose FF (1 mL; GE Healthcare BioScience), followed by transfer to a Superdex 200 10/300 column (GE Healthcare BioScience) to obtain high-purity patatin.

### 3.3. High-Pressure Treatment of Patatin Samples

HHP treatment was performed using a high-pressure device (model HHP.L3-600/0.6; Tianjin Huatai Senmiao Engineering and Technique Co. Ltd., Tianjin, China) and a hydraulic type cell with an inner capacity of 1 L and a water jacket for temperature control. Patatin solution (2%) was prepared in mili-Q water, and 10 mL of each patatin solution was packed in polyethylene bags under vacuum to remove air bubbles. All solutions were then pressure-treated at 25 °C for 15 min at 250 MPa, 350 MPa, 450 MPa, and 550 MPa. The target pressure was reached at a rate of ~250 MPa·min^−1^ and released at ~300 MPa·min^−1^. An unpressurized sample (0.1 MPa) was referred to as NP.

### 3.4. Patatin MW Determination

Patatin MW determination was performed under reducing conditions using one-dimensional sodium dodecyl sulfate polyacrylamide gel electrophoresis (SDS-PAGE; ATTO Corporation, Tokyo, Japan) according the method described by Laemmli et al. [64], with slight modifications. Separating- and stacking-gel recipes were prepared at 12.5% and 5%, respectively, using a discontinuous buffer system. Samples were boiled in loading buffer containing 75 mM Tris-HCl, pH 6.8, 5% (*v*/*v*) β-mercaptoethanol (a thiol-reducing agent), 2% (*w*/*v*) SDS, 10% glycerol, 150 mM EDTA-Na, sucrose (60% (*w*/*v*)), and 0.01% bromophenol blue and denatured at 97 °C in a water bath for 5 min. Each denatured sample (20 μL) along with 8 μL of prestained protein-standard solution (PageRuler prestained protein ladder, 10–170 kDa range; Thermo Fisher Scientific, Waltham, MA, USA) was loaded into each well of a dual-plate electrode assembly with running buffer (25 mM Tris, 192 mM glycine, 0.1% (*w*/*v*) SDS (pH 8.3), and 0.01% (*v*/*v*) β-mercaptoethanol) at 30 mA constant power for ~30 min at room temperature. The electrophoresed gel was transferred to staining solution (0.25% Coomassie Brilliant Blue R-250 in 10% (*v*/*v*) CH_3_COOH and 40% (*v*/*v*) methanol) on a shaker with gentle agitation for 2 h. The gel was destained with 40% (*v*/*v*) methanol and 10% (*v*/*v*) CH_3_COOH, and gel imaging was performed upon appearance of clear electrophoretic band patterns.

### 3.5. Neutral Sugar Composition of Patatin

The monosaccharide composition of NP and HHP-treated patatin was determined using the method of Ogutu and Mu [65], with some modifications. Initially, 5 mg of each sample was hydrolyzed in 4 mL of 4 M trifluoroacetic acid (TFA) at 121 °C for 2 h. After cooling to room temperature, TFA was evaporated in a water bath with continuous nitrogen flushing until dry. Evaporated samples were diluted to 10 mL with Mili-Q water to a concentration of 10 ppm. Individual sugars were quantified by high-performance anion-exchange chromatography (HPAEC-ICS 3000 Dionex System; Thermo Fisher Scientific) with an AS50 Dionex autosampler (3 × 30 mm CarboPac PA20 guard column and 3 × 150 mm CarboPac PA20 column; Thermo Fisher Scientific) connected in series and an ED fluorometer detector (Thermo Fisher Scientific) used to monitor the sugars. Chromatography conditions were employed according to previously described methods [66].

Eluent A consisted of Mili-Q water (0.055 μS∙cm^−1^ at 25 °C), eluent B contained 1 M sodium acetate (NaOAc) in 250 mM NaOH, and eluent C consisted of only 250 mM NaOH. All eluents were filtered through 0.2-μm filter paper (Whatman; GE Healthcare Life Sciences, Little Chalfont, UK) prior to use. Standard solutions containing neutral sugars (l-[−]-arabinose, d-[+]-galactose, d-[+]-xylose, d-[+]-glucose, d-[+]-mannose, and l-rhamnose) with concentration ranges of 0.001 ppm, 0.05 ppm, 0.1 ppm, 0.5 ppm, 1 ppm, and 5 ppm in a single run (35 °C) at a flow rate of 0.5 mL·min^−1^ were used to confirm the linearity of the detector response and to determine the relative response factors. Each sample (10 μL) was injected using a 0.45-µm Ministart filter membrane at the same flow rate as that of the standard.

### 3.6. Fourier Transform Infrared Spectroscopy (FTIR) Spectrum of Patatin

Primary structural changes between NP and HHP-treated patatin were determined using FTIR spectroscopy. Lyophilized samples (NP and HHP-treated) were pulverized with potassium bromide (KBr; 1:100 (*w*/*w*)) and pressed under hydraulic pressure at ~100 bars to obtain an even, clear pallet disk of ~13-mm diameter. Infrared spectra were recorded on an IR spectrophotometer (Tensor-27 spectrometer; Bruker, Bremen, Germany) equipped with an attenuated total reflection ATR system (MKII Golden Gate; Specac, Orpington, UK) and a deuterated triglycine sulfate detector (Nicolet, Thermo Fisher Scientific). Spectra in the range of 4000 cm^−1^ to 400 cm^−1^ were obtained with an average of 64 scans and a resolution of 4 cm^−1^ at room temperature. Each spectrum was baseline-corrected against a blank (KBr only). The absorption peaks were fixed using spectrometer-coupled software OPUS version 6.5 (Bruker).

### 3.7. Differential Scanning Calorimetry (DSC)

The thermal denaturation and stability of NP and HHP-treated patatin were analyzed using a differential scanning calorimeter (Q200; TA Instruments, New Castle, DE, USA) [67]. NP and HHP-treated patatin were accurately weighed (≤1 mg each), followed by transfer to an aluminum pan with 10 μL of 0.05 M phosphate buffer (pH 7.0) and mixing to uniformity. Pans containing slurries were hermetically sealed prior to analysis. The calorimeter was calibrated using an empty pan containing 10 μL of 0.05 M phosphate buffer (pH 7.0) as a reference. Samples were heated at a programmed rate of 20 °C to 95 °C at 5 °C·min^−1^ intervals. The onset (T_on_), peak, denaturation (T_d2_), and off-set (T_off_) temperatures, as well as the enthalpies of denaturation (∆H), were recorded from the thermogram using Universal Analysis 2000 software (TA Instruments).

### 3.8. Circular Dichroism (CD)

CD allows the study of protein stability, folding, and interactions. CD measurements were performed using a MS-450 spectropolarimeter (BioLogic Science Instruments, Grenoble, France). All samples (0.25 mg·mL^−1^) were prepared in 10 mM phosphate buffer (pH 7.0). Far-UV CD spectra were recorded as the average of triplicate scans (190–240 nm) in a 0.1 cm path-length quartz cuvette using a scan speed of 1000 nm·min^−1^, a wavelength step of 1 nm, and a response time of 0.5 s. Input unit was Ɵ in deg·cm^2^·dmol^−1^. All spectra were baseline corrected by subtraction of the spectrum of a protein-free phosphate buffer (10 mM; pH 7.0).

Data were generated by instrument-connected software (Bio-Kine 32 v4.73; BioLogic Science Instruments), and interpretation of CD spectra was performed using the Online Circular Dichroism Analysis program DICHROWEB [68]. The algorithm and reference database used were CONTIN and Set7, respectively.

### 3.9. Surface Hydrophobicity (H_o_)

The H_o_ of NP and HHP-treated patatin was measured using 1-anilino-8-naphthalene sulfonate (ANS) as the hydrophobic fluorescence probe [69]. ANS solution (8 mM) was prepared in 10 mM phosphate buffer (pH 7.0), and protein solutions (4 mL) at various concentrations from 0.005% to 0.025% (*w*/*v*) in 10 mM phosphate buffer (pH 7.0) were thoroughly mixed with 20 mL of freshly prepared ANS. The mixtures were shaken vigorously and stored for 10 min in the dark. The fluorescence intensity (FI) of each sample was measured at 390 nm (excitation), and emission within the range 300 nm to 800 nm was recorded using a fluorometer (F-4500; Hitachi, Tokyo, Japan). Net FI of each solution (FI_Net_) was calculated as:

FI_Net_ = FI of protein dilution blank − FI of protein solution with ANS

The initial slope of FI versus protein concentration (%; *w*/*v*) was calculated using linear regression analysis and used as an index of H_o_.

### 3.10. Determination of Free Sulfhydryl Groups

NP and HHP-treated samples were determined using Ellman’s reagent according to the method of Beveridge et al. [70], with slight modifications. NP and HHP-treated samples (4 mg·mL^−1^) were prepared in 0.086 M tris-glycine buffer (0.09 M glycine, 0.004 M EDTA, and 8 M urea; pH 8.0). An aliquot (3 mL) of the sample was then mixed with 40 µL Ellman’s reagent (4 mg·mL^−1^ of 5,5′-dithiobis-[2-nitrobenzoic acid]) in tris-glycine buffer. The mixture was incubated at room temperature for 30 min, followed by centrifugation at 1000× *g* for 5 min. The absorbance of the mixture was measured at 412 nm using a UV-Vis spectrophotometer, with tris-glycine buffer used as a blank. Results consisted of the absorbance value divided by the molar extinction coefficient of 13,600 mol·L^−1^·cm, and free-sulfhydryl (SH) content was expressed as µmol·g^−1^ of protein. All measurements were conducted in triplicate.

### 3.11. DPPH-Radical-Scavenging Activity Assay

The DPPH-radical-scavenging activity of patatin was measured according to the method described by Zhang et al. [71], with modifications. Each sample (1 mL; 2, 3, and 4 mg·mL^−1^) was added to 2 mL of freshly prepared DPPH solution (0.1 mM in 95% ethanol). The mixture was vortexed using a mixer (WH-2 vortex Mixer; Huxi Analysis Instrument Factory Co., Ltd., Shanghai, China) and incubated in the dark at 27 °C for 30 min. The absorbance of each solution was measured at 517 nm using a UV-vis spectrophotometer (Persee TU-1810 UV-vis; Persee Instruments Co. Ltd., Beijing, China) at room temperature. The lower absorbance of the reaction mixture indicated higher radical-scavenging activity. Radical-scavenging activity was calculated as the percentage of DPPH discoloration using the following equation:

DPPH-radical-scavenging activity % = 100 × [1 − AE/AD]

where “AE” represents the solution absorbance at 517 nm when 1 mL of each patatin solution was mixed with 2 mL of 0.1 mmol·L^−1^ DPPH solution after incubation (30 min) at room temperature, and “AD” represents the absorbance of 2 mL of 0.1 mmol·L^−1^ DPPH solution with 1 mL Milli-Q H_2_O.

### 3.12. Oxygen-Radical Absorbance Capacity (ORAC) Assay

An ORAC assay was performed according to the method described by Prior et al. [72]. Phosphate buffer (0.075 M; pH 7.4) was used as a diluent to prepare all reagents and samples. Briefly, sample solutions (20 μL) at different concentrations (1, 2, 3 and 4 mg·mL^−1^) were added to 20 μL phosphate buffer in 96-well microtiter plates. Subsequently, 20 μL sodium fluorescein solution (63 nmol·L^−1^) was added to each well, followed by incubation at 37 °C for 15 min. After incubation, 140 μL of freshly prepared AAPH (18.28 mmol·L^−1^) solution was pipetted into the duplicate wells, whereas other duplicate wells were diluted with 140 μL of phosphate buffer (0.075 M; pH 7.4) alone. After 10 min of vigorous shaking, fluorescence intensity was measured using a ChameleonTM V multi-label microplate reader (Hidex, Turku, Finland). The system was set to fluorescence mode, and the fluorescence intensity of each well was recorded 60 times at 2-min intervals. The excitation and emission filter wavelengths were set at 485 nm and 535 nm, and the detection temperature was kept constant at 37 °C.

The fluorescence intensity of each sample was determined without the effect of AAPH (i.e., AAPH solution was replaced by an equal amount of phosphate buffer) in order to calculate the relative fluorescence intensity using Equation (1). The relative fluorescence intensity was used to calculate the area under the curve (AUC) using the approximate integration method shown in formula (2). ORAC values were expressed as the net area under the curve (netAUC) between the samples and the blank as shown in Equation (3). A calibration curve for the trolox standards (at concentrations of 5, 10, 20, 40 and 60 μg·mL^−1^) was prepared. The linear regression equation was *y* = 0.8898*x* + 2.5805 (R^2^ = 0.9929). ORAC values of the samples were expressed as μg Trolox equivalent per 100 g of sample (μg TE 100 g^−1^). The results were interpreted according to the ORAC equation as follows:

(1) $$F_{i}=\mathrm{fi}\left(+\mathrm{AAPH}\right)÷\mathrm{fi}\left(-\mathrm{AAPH}\right)$$

(2) $$\mathrm{AUC}=\;\Delta t\times F_{0}+F_{1}\ldots \ldots \ldots +F_{n}$$

(3) $$\mathrm{net}\;\mathrm{AUC}={\mathrm{AUC}}_{\mathrm{sample}}-{\mathrm{AUC}}_{\mathrm{blank}}$$

where fi(+AAPH) represents the fluorescence intensity of the reaction solution containing the AAPH solution, fi(−AAPH) represents the fluorescence intensity of the reaction solution without AAPH, and F_i_ represents the relative fluorescence intensity of the reaction solution. The AUC corresponds to the relative fluorescence decay, and (Δt) represents the interval time, with Δt in this study at 2 h. AUC_sample_ and AUC_blank_ represent the AUCs of the sample and the blank, respectively, and net AUC represents the net AUC between the sample and the blank.

### 3.13. Ferrous Ion-Chelating Activity

Determination of ferrous ion-chelation activity was performed according to the method of Miao et al. [73] and at different concentrations of NP and HHP-treated patatin (1, 2, 3 and 4 mg·mL^−1^). Each sample (600 µL) was mixed with freshly prepared FeSO_4_ (60 µL) and 2.42 mL Milli-Q H_2_O and incubated for 30 min at room temperature with continuous shaking (80 rpm). After incubation, 120 µL of 5 mM ferrozine solution was added to the mixture, and decolorization due to Fe^2+^ dissociation was monitored by determining the absorbance at 532 nm. Milli-Q H_2_O and EDTA were used as negative and positive controls, respectively. The percentage of inhibition of ferrozine–Fe^2+^ complex formation was determined using the following formula:

(4) $$\mathrm{Iron}\;\left(\mathrm{II}\right)\;\mathrm{Chelating}\;\mathrm{activity}\;\left(\%\right)=\left[\frac{\left(\mathrm{Blank}\;\mathrm{absorbance}\;–\;\mathrm{Sample}\;\mathrm{absorbance}\right)}{\mathrm{Blank}\;\mathrm{absorbance}}\right]\times 100$$

### 3.14. Statistical Analysis

Results were expressed as the mean ± standard deviation (SD) of triplicate experiments unless otherwise stated. GraphPad Prism software version 5.0 (GraphPad Software; San Diego, CA, USA) and SAS 8.1 software (SAS Institute, Cary, NC, USA) were used for all statistical analyses and to generate data plots. OriginPro 8.5.1 (Origin Lab Corporation, Wellesley Hills, MA, USA) was used to plot FTIR spectra. Statistical significance was determined at *p* < 0.05.

## 4. Conclusions

Patatin was effectively isolated and purified from PFJ and modified by HHP treatment (250–550 MPa). The effects of HHP treatment on the structure, composition, heat profile, and antioxidant potential indicated significant changes in patatin structure and physical properties, which enhanced its physicochemical behavior. HHP treatment also enhanced the antioxidant potential of patatin according to results measuring DPPH-radical-scavenging ability and ORAC. Iron-chelation activity was also improved significantly (*p* < 0.05) along with increases in HHP treatment. Furthermore, we observed significant enhancement of antioxidant potential in HHP-treated patatin as compared with that observed in the NP. Our findings suggested that pressurization might significantly contribute to patatin stabilization. The structural, thermal, and bio-functional properties were dependent upon the intensity of HHP treatment. Therefore, this suggested that patatin from *S. tuberosum* L. might constitute a potential antioxidant for use in enhancing human health. Moreover, our findings revealed the potential application of HHP treatment as an economical and effective means of modification that might contribute to the conversion of certain proteins into value-added ingredients. Despite this progress, there remain many unanswered questions and unexplained avenues of research to be explored.

## Figures and Tables

**Table 1:** Effect of HHP treatment on the monosaccharide content of patatin. Values with different letters within the same column signify significant differences (*p* < 0.05).

Treatments	Rhamnose	Galactose	Glucose	Xylose	Mannose
NP	0.97 ± 0.01 ^c^	14.15 ± 0.64 ^d^	14.30 ± 0.25 ^a^	0.08 ± 0.00 ^a^	0.445 ± 0.02 ^b^
250 MPa	1.69 ± 0.05 ^a^	18.04 ± 0.57 ^a^	10.93 ± 0.24 ^d^	0.08 ± 0.00 ^a^	0.563 ± 0.02 ^a^
350 MPa	0.82 ± 0.03 ^d^	12.16 ± 0.17 ^e^	10.70 ± 0.08 ^e^	0.07 ± 0.00 ^b,c^	0.422 ± 0.01 ^b^
450 MPa	0.97 ± 0.02 ^c^	14.27 ± 0.61 ^c^	12.30 ± 0.25 ^b^	0.07 ± 0.00 ^a,b^	0.467 ± 0.00 ^b^
550 MPa	1.40 ± 0.03 ^b^	17.03 ± 1.13 ^b^	11.24 ± 0.37 ^c^	0.06 ± 0.00 ^c^	0.463 ± 0.01 ^b^

**Table 2:** DSC thermograms of NP and HHP-induced patatin.

Sample	T_on-set_ (°C)	T_d_ (°C)	ΔT_(1/2)_	ΔH (J·g^−1^)
NP	58.89 ± 0.48 ^c^	66.62 ± 0.61 ^a^	5.84 ± 0.98 ^b,c^	24.03 ± 1.93 ^a^
250 MPa	60.06 ± 0.27 ^a^	65.98 ± 0.28 ^b,c^	5.32 ± 1.98 ^d^	12.56 ± 2.32 ^b^
350 MPa	58.53 ± 0.32 ^c^	65.88 ± 0.37 ^b^	5.56 ± 0.76 ^c^	6.97 ± 1.53 ^c^
450 MPa	59.99 ± 0.81 ^b^	65.88 ± 0.54 ^b^	7.07 ± 0.58 ^a^	5.72 ± 2.06 ^d^
550 MPa	60.41 ± 0.42 ^a^	65.64 ± 0.31 ^b,c^	6.04 ± 0.98 ^b^	3.05 ± 0.32 ^e^

Onset temperatures (T_on-set_), peak or denaturation temperatures (T_d_), and enthalpies of denaturation (∆H). Values with different letters in the same column are significantly different according to Duncan’s multiple range test (*p* < 0.05).

**Table 3:** CD results of NP and HHP-treated patatin.

Sample	α-Helix	β-Strand	β-Turn	Random Coil
NP	24.2 ^a^	24.2 ^e^	21.2 ^a^	30.3 ^d^
250 MPa	21.7 ^b^	26.5 ^d^	21.0 ^a^	30.9 ^c,d^
350 MPa	16.3 ^c^	31.6 ^c^	20.6 ^b^	31.4 ^c^
450 MPa	7.3 ^d^	36.4 ^b^	21.4 ^a^	34.8 ^b^
550 MPa	4.1 ^e^	39.5 ^a^	19.2 ^c^	37.2 ^a^

Values with different letters in the same column are significantly different according to Duncan’s multiple range test (*p* < 0.05).
